# Psychological rehabilitation of women with endometrial cancer: dynamics of quality of life after psychotherapeutic intervention depending on the type of surgical treatment

**DOI:** 10.3389/fpsyg.2026.1917674

**Published:** 2026-08-19

**Authors:** Ayazhan Janbayeva, Aliya Uteulova, Piko Bettina, Marat Assimov, Fatima Bagiyarova

**Affiliations:** 1 Higher School of Psychology, Turan University, Almaty, Kazakhstan; 2 Department of Psychological and Social Support, Kazakh Institute of Oncology and Radiology, Almaty, Kazakhstan; 3 University of Szeged, Szeged, Hungary; 4 Asfendiyarov Kazakh National Medical University, Almaty, Kazakhstan

**Keywords:** art therapy, body-oriented psychotherapy, endometrial cancer, oncology, psychology, quality of life, rehabilitation, surgical treatment

## Abstract

Endometrial cancer is the most common gynecological malignancy among women, and its incidence has increased considerably over recent decades. Surgical treatment, chemotherapy, radiotherapy, and hormone therapy are frequently associated with psychological distress, impaired social functioning, and reduced quality of life. The present study aimed to examine changes in quality of life among women with endometrial cancer across different stages of treatment and rehabilitation, as well as to assess changes associated with a psychotherapeutic intervention combining individual and group psychological counseling with art therapy and body-oriented psychotherapy techniques. The study included 40 women diagnosed with endometrial cancer who completed the WHOQOL-BREF questionnaire at three time points: before treatment, after completion of the main treatment, and during the rehabilitation period. Treatment-related factors, including the type of surgery (laparoscopic total hysterectomy, radical hysterectomy, or no surgery), chemotherapy, radiotherapy, and hormone therapy, were also evaluated. Statistical analysis demonstrated significant improvements in self-perception (*p* < 0.001) and social wellbeing (*p* = 0.002) during the rehabilitation period compared with the intermediate stage of treatment, while physical and psychological wellbeing remained relatively stable. Patients who did not receive adjuvant chemotherapy showed significantly better quality-of-life outcomes during the intermediate stage, whereas these differences largely diminished over time. During rehabilitation, women who underwent laparoscopic total hysterectomy demonstrated higher levels of self-perception and microsocial support, while the absence of hormone therapy was associated with better self-perception and social wellbeing. These findings demonstrate changes in quality-of-life indicators across the treatment and rehabilitation trajectory in women with endometrial cancer. Improvements in self-perception and social functioning were observed during rehabilitation; however, because the study did not include a control group, these changes cannot be attributed specifically to the psychotherapeutic intervention. Natural recovery, adaptation to the disease, completion of active treatment, and treatment-related factors may also have contributed to the observed dynamics. The results support the need for larger controlled studies of integrative psycho-oncological rehabilitation programs. Because subgroup sizes were small and multiple exploratory comparisons were performed, treatment-related findings should be interpreted as preliminary.

## Introduction

Over the past decades, the incidence of endometrial cancer has increased considerably, making it the most common gynecological malignancy among women (Sung et al., 2021; Siegel et al., 2025). Despite substantial advances in diagnosis and treatment, preserving patients' quality of life has become an important clinical challenge, particularly given the growing proportion of younger women affected by the disease (Concin et al., 2021).

Current management of endometrial cancer involves various combinations of surgical treatment, chemotherapy, radiotherapy, and hormone therapy. Although these approaches have improved oncological outcomes, they may also be associated with chronic pain, physical limitations, altered body image, sexual dysfunction, anxiety and depressive symptoms, reduced self-esteem, and impaired social functioning (Bowes et al., 2014; Blinov et al., 2023). The severity of these consequences may depend not only on disease characteristics but also on treatment-related factors, including the extent of surgery and the use of adjuvant therapies.

Health-related quality of life (HRQoL) has emerged as an important outcome measure in gynecological oncology. Patient-reported outcomes (PROs) provide valuable information regarding the physical, psychological, and social consequences of cancer and its treatment and contribute to a more comprehensive evaluation of rehabilitation needs (Basch, 2017; Himpe et al., 2026).

Accumulating evidence suggests that women with gynecological malignancies require multidisciplinary psychosocial support throughout treatment and rehabilitation. However, the impact of different treatment modalities for endometrial cancer on quality of life and psychological wellbeing, as well as the potential role of psychotherapeutic interventions in improving these outcomes, remains insufficiently investigated (Bowes et al., 2014; Himpe et al., 2026).

Therefore, the aim of the present prospective longitudinal study was to describe changes in quality of life among women with endometrial cancer across different stages of treatment and rehabilitation. A secondary aim was to explore whether quality-of-life indicators differed according to treatment-related factors, including type of surgical intervention, chemotherapy, radiotherapy, and hormone therapy. Because the study did not include a control group, it was not designed to establish the causal effectiveness of the psychotherapeutic intervention.

## Methods

As part of the psychotherapeutic component of the study, a structured psychological intervention program was implemented before treatment, after completion of treatment, and during the rehabilitation period. The program was designed to reduce emotional distress, enhance self-perception, and improve psychosocial adaptation among women diagnosed with endometrial cancer. Body-oriented psychotherapy techniques were applied in combination with art therapy methods.

The psychotherapeutic intervention and quality-of-life assessment procedures followed the standardized protocol previously developed and described by our research group (Uteulova et al., 2026). The present study applied the same intervention schedule and assessment methodology while additionally evaluating the influence of treatment-related factors, including the type of surgery, chemotherapy, radiotherapy, and hormone therapy, on quality-of-life outcomes in women with endometrial cancer. The study was designed as a prospective longitudinal quasi-experimental study with repeated measures to assess changes in quality-of-life indicators at three time points. The study used a single-group repeated-measures design and did not include a control or comparison group. Therefore, the design allowed the assessment of within-participant changes over time but did not permit causal attribution of these changes to the psychotherapeutic intervention. The psychological intervention program was administered at three stages:

• Prior to the initiation of medical treatment (baseline assessment);

• Immediately after completion of the main treatment (post-treatment assessment);

• During the rehabilitation phase, approximately 21 days after treatment completion (follow-up assessment).

A total of 40 women with histologically confirmed endometrial cancer participated in the study. Patients received treatment at an oncological medical center and underwent standard diagnostic and therapeutic procedures for endometrial cancer.

Completion of the treatment phase included standard oncological management according to clinical indications, including surgical treatment, chemotherapy, radiotherapy, and hormone therapy. Surgical management comprised laparoscopic total hysterectomy, radical hysterectomy, or no surgical intervention.

### Inclusion criteria

The inclusion criteria for participation in the study were as follows:

• Women aged 28–78 years with histologically confirmed endometrial cancer;

• Provision of written informed consent;

• Patients undergoing treatment and rehabilitation for endometrial cancer.

### Non-inclusion criteria

Participants were not included in the study if they met any of the following criteria:

• Absence of a confirmed diagnosis of endometrial cancer;

• Incomplete case report form (CRF) data;

• Lack of informed consent;

• Diagnosed severe psychiatric disorders interfering with participation.

### Exclusion criteria

Participants were excluded from the study under the following conditions:

• Withdrawal from the study at the participant's request;

• Change of country or region of residence during the study period;

• Death before completion of the study.

A total of 40 eligible patients were invited to participate, and all provided informed consent and completed the three assessments. No participants withdrew from the study, and no deaths or losses to follow-up occurred during the observation period.

Quality of life was assessed using the Russian-language version of the WHOQOL-BREF questionnaire. The instrument consists of 26 items rated on a five-point Likert scale.

In the present study, items were grouped into four domains:

• Physical and psychological wellbeing;

• Self-perception;

• Microsocial support;

• Social wellbeing.

The domain grouping used in the present analysis followed the scoring structure of the validated Russian-language adaptation applied by our research group.

Reverse coding was applied for negatively worded items before score calculation. Domain scores were calculated as the sum of the corresponding item responses.

The psychotherapeutic intervention consisted of three structured sessions conducted throughout the treatment trajectory. One session was delivered at each assessment stage and lasted approximately 60–90 min.

Sessions were conducted individually or in small groups of four to six participants by qualified clinical psychologists experienced in psycho-oncological care. The intervention integrated body-oriented psychotherapy techniques with expressive art therapy methods.

Each session included three consecutive components. The first component involved somatic regulation techniques, including diaphragmatic breathing, grounding exercises, guided body scanning, and muscle relaxation practices based on body-oriented psychotherapy approaches. The second component included expressive art therapy activities such as drawing, collage making, and body-mapping aimed at facilitating symbolic expression of illness-related experiences. The third component consisted of verbal reflection and group discussion designed to integrate emotional, bodily, and cognitive aspects of the participants' experiences.

At each assessment point, participants completed the WHOQOL-BREF questionnaire before the psychotherapeutic session scheduled for that stage. Thus, the baseline assessment preceded the first session, the post-treatment assessment preceded the second session, and the rehabilitation assessment preceded the third session. Consequently, the third assessment could reflect changes occurring after the first two sessions, completion of medical treatment, natural recovery, and psychosocial adaptation, but it did not capture the immediate or subsequent effect of the third session. The present study, therefore, evaluated longitudinal changes occurring alongside the intervention program rather than the isolated causal effect of each psychotherapeutic session.

All 40 participants attended all three scheduled psychotherapeutic sessions and completed the assessments at each time point. Therefore, there was no intervention-related attrition among the participants included in the final analysis.

In addition to evaluating longitudinal changes in quality of life, the study examined the influence of treatment-related factors, including the type of surgical intervention, chemotherapy, radiotherapy, and hormone therapy, on quality-of-life outcomes during treatment and rehabilitation.

No formal a priori sample-size calculation was performed because the study was exploratory and recruitment was limited by the number of eligible patients treated during the study period. Consequently, subgroup analyses may have been underpowered, particularly for radical hysterectomy and hormone therapy groups.

## Results

### Clinical and demographic characteristics of the study population

The study included 40 women diagnosed with endometrial cancer. The mean age of the participants was 57.2 years (SD = 13.7), ranging from 28 to 78 years. Age distribution did not significantly deviate from normality according to the Kolmogorov-Smirnov test (*p* = 0.138).

The majority of patients were older than 60 years (52.5%, *n* = 21), followed by women aged 31–40 years (15.0%, *n* = 6), 41–50 years (10.0%, *n* = 4), and 51–60 years (10.0%, *n* = 4). One participant (2.5%) was younger than 30 years. Regarding surgical treatment, laparoscopic total hysterectomy was performed in 70.0% of patients (*n* = 28), whereas radical hysterectomy was performed in 20.0% (*n* = 8).

Adjuvant chemotherapy was administered to 40.0% of patients (*n* = 16), whereas 60.0% (*n* = 24) did not receive chemotherapy.

Radiotherapy was performed in 60.0% of patients (*n* = 24), while 40.0% (*n* = 16) did not receive radiation treatment.

Hormone therapy was administered to 22.5% of patients (*n* = 9), whereas the majority of participants (77.5%, *n* = 31) did not receive hormonal treatment.

Clinical and demographic characteristics were summarized using frequencies and percentages for categorical variables and means (M), standard deviations (SD), medians (Me), and interquartile ranges (Q1–Q3) for continuous variables.

Changes in quality-of-life indicators across the three assessment points were evaluated using the Friedman test for repeated measures, followed by pairwise comparisons with the Wilcoxon signed-rank test. Bonferroni correction was applied for multiple comparisons, with the significance threshold adjusted to α = 0.017 (0.05/3). To improve the accuracy of the estimates, Monte Carlo simulations with 10,000 samples and 95% confidence intervals (CIs) were used. Effect sizes were calculated as *r* = *Z*/√*N*.

At the nominal *p* < 0.05 level, several treatment-related associations were observed; however, these findings were not adjusted for multiple comparisons and should be considered exploratory.

Physical and psychological wellbeing did not show statistically significant overall changes across the three assessment points (Friedman test, *p* = 0.075; Table 1). Although the Stage 3 vs. Stage 2 comparison yielded *p* = 0.014 and a moderate effect size (*r* = 0.39), the overall Friedman test was not statistically significant. Therefore, this pairwise finding should be considered exploratory rather than evidence of a confirmed longitudinal change.

**Table 1:** Dynamics of quality-of-life indicators across the three assessment points in the total sample (*n* = 40).

Quality-of-life domain	Friedman test, *p*	Significant pairwise comparisons	Effect size (*r*)
Physical and psychological wellbeing	0.075	Stage 3 > Stage 2 (*p* = 0.014)	0.39
Self-perception	0.002	Stage 3 > Stage 2 (*p* < 0.001)	0.56
Microsocial support	0.012	No significant pairwise differences after Bonferroni correction (closest: Stage 3 vs. Stage 1, *p* = 0.029)	–
Social wellbeing	0.007	Stage 3 > Stage 1 (*p* = 0.001)	0.54
		Stage 3 > Stage 2 (*p* = 0.002)	0.5

Self-perception demonstrated significant longitudinal changes (Friedman test, *p* = 0.002). Patients reported significantly better self-perception during rehabilitation than immediately after treatment (*p* < 0.001, *r* = 0.56), corresponding to a large effect size. This pattern is consistent with improved self-perception during rehabilitation, although the contribution of the psychotherapeutic intervention cannot be separated from natural recovery or completion of medical treatment.

Microsocial support also changed significantly over time (Friedman test, *p* = 0.012). Although pairwise comparisons did not remain significant after Bonferroni correction, the observed tendency toward higher scores during rehabilitation may indicate gradual improvement in perceived social support and interpersonal functioning.

Social wellbeing demonstrated the most pronounced positive dynamics (Friedman test, *p* = 0.007). Scores during the rehabilitation period were significantly higher than both baseline (*p* = 0.001, *r* = 0.54) and post-treatment assessments (*p* = 0.002, *r* = 0.50), with large effect sizes. These findings suggest that social wellbeing scores were higher during the rehabilitation assessment than at the preceding assessments.

Overall, the results indicate that the rehabilitation period is associated with the most favorable quality-of-life outcomes. While physical and psychological wellbeing remained relatively stable, significant improvements in self-perception and social wellbeing, together with positive trends in microsocial support, suggest progressive psychosocial adaptation throughout treatment and rehabilitation in women with endometrial cancer.

To investigate the potential influence of treatment-related factors on quality-of-life outcomes, subgroup analyses were performed at the second (post-treatment) and third (rehabilitation) assessment points. Comparisons were conducted according to the type of surgical intervention, administration of adjuvant chemotherapy, radiotherapy, and hormone therapy.

Patients were classified into the following treatment groups:

- Type of surgery: laparoscopic total hysterectomy (*n* = 28) vs. radical hysterectomy (*n* = 8);
- Chemotherapy: no chemotherapy (*n* = 22) vs. adjuvant chemotherapy following surgery (*n* = 16);
- Radiotherapy: no radiotherapy (*n* = 16) vs. radiotherapy (*n* = 24);
- Hormone therapy: no hormone therapy (*n* = 31) vs. hormone therapy (*n* = 9).

Patients who did not undergo surgery (*n* = 4) were excluded from the comparison of surgical techniques because meaningful comparison was only possible between the two surgical procedures.

Although 24 patients did not receive chemotherapy, quality-of-life data at the second assessment point were available for 22 patients because two questionnaires contained missing values.

The effects of these treatment modalities on physical and psychological wellbeing, self-perception, microsocial support, and social wellbeing were evaluated at both assessment points to identify potential differences in quality-of-life outcomes associated with the therapeutic approach.

At the second assessment point, adjuvant chemotherapy was the only treatment-related factor significantly associated with quality-of-life outcomes (Table 2). Patients who did not receive adjuvant chemotherapy reported significantly better physical and psychological wellbeing (*U* = 87.0, *p* = 0.008), self-perception (*U* = 78.0, *p* = 0.004), and social wellbeing (*U* = 100.5, *p* = 0.025) than those who underwent adjuvant chemotherapy. A trend toward higher self-perception was observed according to the type of surgical intervention (*p* = 0.060), while a trend toward lower physical and psychological wellbeing was observed among patients receiving hormone therapy (*p* = 0.071). Neither finding reached the conventional threshold for statistical significance.

**Table 2:** Comparison of quality-of-life indicators according to treatment modalities at the second assessment point.

Quality-of-life domain	Type of surgery (*p*)	Chemotherapy (*p*)	Radiotherapy (*p*)	Hormone therapy (*p*)
Physical and psychological wellbeing	0.117	**0.008** ^*^	0.145	0.071
Self-perception	0.060	**0.004** ^*^	0.550	0.304
Microsocial support	0.619	0.149	0.396	0.781
Social wellbeing	0.109	**0.025** ^*^	0.740	0.173

*p < 0.05. Bold values indicate statistically significant results (*p* < 0.05).

At the third assessment point, several treatment-related factors were associated with quality-of-life outcomes (Table 3).

**Table 3:** Differences in quality-of-life outcomes according to treatment-related factors at the third assessment point.

Quality-of-life domain	Type of surgery (*p*)	Chemotherapy (*p*)	Radiotherapy (*p*)	Hormone therapy (*p*)
Physical and psychological wellbeing	0.249	0.822	0.219	0.350
Self-perception	**0.003** ^*^	0.364	0.337	**0.007** ^*^
Microsocial support	**0.017** ^*^	0.044^*^	0.041^*^	0.070
Social wellbeing	0.056	0.182	0.193	**0.042** ^*^

*p < 0.05. Bold values indicate statistically significant results (*p* < 0.05).

Patients who underwent laparoscopic total hysterectomy demonstrated significantly higher self-perception (*U* = 33.5, *p* = 0.003) and microsocial support (*U* = 50.0, *p* = 0.017) compared with those who underwent radical hysterectomy. In addition, a tendency toward better social wellbeing was observed in the laparoscopic surgery group, although the difference did not reach statistical significance (*p* = 0.056).

Hormone therapy was also associated with quality-of-life outcomes during the rehabilitation period. Patients who did not receive hormone therapy reported significantly higher self-perception (*U* = 56.5, *p* = 0.007) and social wellbeing (*U* = 77.0, *p* = 0.042) than those who received hormonal treatment.

The impact of chemotherapy was less pronounced at the third assessment point than at the post-treatment stage. Most quality-of-life differences between groups were no longer statistically significant; however, patients who did not receive adjuvant chemotherapy continued to demonstrate higher levels of microsocial support (*U* = 109.0, *p* = 0.044).

Similarly, radiotherapy was associated with microsocial support during the rehabilitation period. Patients who did not undergo radiotherapy demonstrated significantly higher microsocial support scores than those who received radiation treatment (*U* = 119.0, *p* = 0.041).

Multivariable analyses were not performed because the total sample and several treatment subgroups were too small to support stable adjusted models without a substantial risk of overfitting. Therefore, the reported treatment-related associations are unadjusted and may be influenced by disease stage, age, comorbidities, socioeconomic factors, or treatment intensity.

Overall, the findings suggest that treatment-related factors, particularly the extent of surgical intervention and the use of hormone therapy, may influence specific domains of quality of life during rehabilitation in women with endometrial cancer.

The study demonstrated that the post-treatment period (the second assessment point) represented the most vulnerable stage for women with endometrial cancer, characterized by the lowest levels of self-perception and physical and psychological wellbeing. By the third assessment point (the rehabilitation phase), significant improvements were observed in self-perception and social wellbeing. These positive changes may reflect gradual adaptation to the disease, completion of active treatment, and progressive reintegration into everyday life.

## Discussion

The present study investigated changes in quality-of-life indicators among women with endometrial cancer during different stages of treatment and rehabilitation and evaluated the influence of treatment-related factors on psychosocial adaptation. The findings contribute to the growing body of evidence emphasizing the importance of comprehensive psycho-oncological rehabilitation and the consideration of treatment characteristics when assessing long-term patient outcomes.

The results demonstrated distinct patterns of change across the treatment trajectory. The post-treatment period (the second assessment point) appeared to represent the most vulnerable stage for women with endometrial cancer, characterized by the lowest levels of self-perception and physical and psychological wellbeing. By the rehabilitation stage, significant improvements were observed in self-perception and social wellbeing, while microsocial support demonstrated a positive trend These findings may reflect gradual adaptation to the disease, completion of active treatment, progressive reintegration into everyday life, and exposure to the psychotherapeutic program. However, because no control group was included and the assessments coincided with major changes in medical treatment status, the independent contribution of psychotherapy cannot be determined.

An important finding of the present study was the differential influence of treatment-related factors on quality-of-life outcomes. Adjuvant chemotherapy demonstrated the strongest association with impaired quality of life during the post-treatment period. Patients receiving chemotherapy reported significantly lower physical and psychological wellbeing, self-perception, and social wellbeing compared with those who did not receive chemotherapy. By the rehabilitation stage, most of these differences had diminished, although lower microsocial support remained among patients who had undergone chemotherapy. These findings may reflect the cumulative physical and emotional burden of systemic treatment and its gradual resolution over time.

The type of surgical intervention also influenced psychosocial outcomes during rehabilitation. Although no significant differences were observed immediately after treatment, patients who underwent laparoscopic total hysterectomy demonstrated better self-perception and microsocial support than those who underwent radical hysterectomy. A tendency toward better social wellbeing was also observed in the laparoscopic surgery group. These findings suggest that less extensive surgical approaches may facilitate more favorable long-term psychological and social adjustment.

Hormone therapy was associated with lower self-perception and social wellbeing during the rehabilitation period. These differences may be related to treatment-associated adverse effects, including vasomotor symptoms, changes in body weight, reduced libido, and other factors that may negatively influence body image and social functioning. In contrast, radiotherapy was not associated with significant differences in most quality-of-life domains, although patients who did not receive radiotherapy demonstrated higher levels of microsocial support during rehabilitation.

Treatment allocation was based on clinical indications rather than randomization. Consequently, the associations between treatment modalities and quality-of-life outcomes may partly reflect differences in disease stage, prognosis, comorbidity burden, or overall treatment intensity. These potential confounders were not controlled statistically and should be considered when interpreting subgroup differences.

Several longitudinal and between-group comparisons yielded moderate to large statistical effect sizes. These estimates indicate that the observed differences may be relevant; however, clinical significance cannot be established because validated minimal clinically important difference thresholds were not applied, confidence intervals for the effect sizes were not reported, and subgroup estimates were based on small samples.

The integration of a psychotherapeutic program combining body-oriented psychotherapy and art therapy constituted an important component of the rehabilitation process. Body-oriented techniques may facilitate emotional regulation through increased awareness of bodily sensations and reduction of somatic tension, whereas art therapy provides opportunities for symbolic expression of illness-related experiences and reconstruction of a positive self-image. Together, these approaches may support emotional processing, psychological resilience, and social adaptation during recovery.

Previous studies have demonstrated that art therapy interventions can reduce emotional distress and improve psychological wellbeing in oncology patients (Monti et al., 2006; Bosman et al., 2020). Similarly, body-oriented psychotherapy approaches have been associated with improved emotional regulation and reduced somatic tension (Röhricht, 2009). The observed changes are compatible with the potential value of integrating creative and body-oriented approaches into psycho-oncological care. Nevertheless, the single-group design does not allow these changes to be distinguished from spontaneous adaptation, completion of treatment, nonspecific therapeutic support, or other concurrent influences.

### Clinical implications

The findings highlight the importance of multidisciplinary rehabilitation for women with endometrial cancer. Particular attention may be warranted for patients undergoing adjuvant chemotherapy, radical surgical treatment, and hormone therapy, as these treatment modalities were associated with less favorable quality-of-life outcomes during recovery. These findings may help identify groups that could benefit from intensified psychosocial assessment and support. Controlled studies are required to determine whether targeted psychological interventions improve outcomes in these subgroups.

### Limitations

Several limitations should be considered when interpreting the findings. First, the study included a relatively small single-center sample, and some treatment subgroups were particularly small. This limited statistical power, increased the risk of type II errors, and reduced the precision and generalizability of subgroup estimates. No formal a priori sample-size calculation was performed.

Second, the study used a single-group quasi-experimental design without a control or comparison group. Therefore, the observed longitudinal changes cannot be attributed specifically to the psychotherapeutic intervention. Natural recovery, adaptation to the diagnosis, completion of active oncological treatment, regression to the mean, and other forms of psychosocial support may have contributed to the findings.

Third, treatment groups were formed according to clinical indications rather than random allocation. The analyses were not adjusted for potential confounding variables such as disease stage, age, comorbidities, socioeconomic status, baseline symptom severity, treatment duration, and overall treatment intensity. Multivariable analyses were not performed because the sample size was insufficient to support stable adjusted models.

Fourth, multiple exploratory Mann–Whitney *U*-tests were conducted across treatment modalities, quality-of-life domains, and assessment points. Because no formal adjustment was applied to these subgroup comparisons, the risk of false-positive findings was increased, particularly for *p*-values close to 0.05. These associations should, therefore, be regarded as preliminary and hypothesis-generating.

Fifth, the assessment schedule did not permit isolation of the effects of individual psychotherapeutic sessions. Questionnaires were administered before the session at each assessment stage, and the final assessment, therefore, did not capture the effect of the third session. Information on intervention adherence, missed sessions, participant attrition, and exposure to additional psychological support should also be considered when evaluating the intervention.

Finally, clinical significance was not assessed using established minimal clinically important difference thresholds, and confidence intervals for effect-size estimates were not reported. Further multicenter controlled studies with larger samples, prespecified hypotheses, adjusted statistical models, longer follow-up, and detailed intervention fidelity assessment are required.

## Conclusion

The present study demonstrated that quality-of-life outcomes in women with endometrial cancer change throughout the course of treatment and rehabilitation and may be influenced by treatment-related factors. The post-treatment period represented the most vulnerable stage, characterized by the lowest levels of self-perception and physical and psychological wellbeing, whereas significant improvements in self-perception and social wellbeing were observed during rehabilitation.

Among the treatment modalities evaluated, adjuvant chemotherapy was associated with less favorable quality-of-life outcomes during the post-treatment period, while laparoscopic total hysterectomy was associated with better self-perception and microsocial support during rehabilitation. Hormone therapy was associated with lower self-perception and social wellbeing, whereas radiotherapy showed only limited associations with quality-of-life domains.

The findings indicate that quality-of-life indicators changed across the treatment and rehabilitation trajectory and that several unadjusted associations with treatment modalities were observed. Because the subgroup analyses were exploratory, involved small samples, and were not adjusted for multiple comparisons or confounding factors, these results should be regarded as preliminary.

Improvements in self-perception and social wellbeing occurred during the period in which participants received integrative psychological support. However, the absence of a control group prevents attribution of these improvements specifically to the intervention. Larger controlled studies are needed to distinguish the effects of psychotherapy from natural recovery, completion of oncological treatment, and other concurrent influences.

## Data Availability

The raw data supporting the conclusions of this article will be made available by the authors, without undue reservation.
